# Rubella outbreak in the school children, Addis Ababa, Ethiopia: February–April 2018

**DOI:** 10.1186/s12879-019-3873-y

**Published:** 2019-03-18

**Authors:** Getachew Dinede, Abigiya Wondimagegnehu, Fikre Enquselassie

**Affiliations:** 1 Epidemiology Directorate, Ministry of Agriculture, Addis Ababa, Ethiopia; 2 Epidemiology Unit, Department of Preventive Medicine, School of Public Health, Addis Ababa University, Addis Ababa, Ethiopia; 3 Head of Department of Preventive Medicine, School Of Public Health, Addis Ababa University, Addis Ababa, Ethiopia

**Keywords:** Descriptive epidemiology, Disease outbreaks, Rubella, School

## Abstract

**Background:**

Rubella is a vaccine-preventable contagious disease causing an estimated 100,000 children to be born with congenital rubella syndrome each year globally. Studies documented that 18 rubella outbreaks were occurred each year in Ethiopia. Yeka sub-city woreda 13 public health emergency management office reported two measles suspected cases on 8 February, 2018. We investigated this outbreak to identify its etiology, describe the outbreak and implement control measures.

**Methods:**

We described the outbreak using descriptive epidemiology. The study population was defined as students learning in the school where the outbreak occurred. Suspected rubella case was defined as student with generalized rash whereas confirmed case was suspected case tested positive for rubella IgM. Questionnaires, checklists and students record review were used to collect data. We searched for new cases in classes daily and excluded them from classes. The school environment was assessed and the outbreak was described in person and time.

**Results:**

We identified 58 cases (median age: 4.6 years; IQR: 4–5 years) with six of them rubella IgM positive and 52 epidemiologically linked. The outbreak began on 8 February 2018 having multiple intermittent peaks during its course reaching its highest peak at 2 April, 2018 and ended on 20 April, 2018. Index cases were reported from two classes; however, cases were occurred in 13/15(86.67%) of the classes during the entire outbreak. Fifty five percent (32/58) and 45/58(77.59%) of the cases were females and 3–5 years children, respectively. Overall attack rate was 58/531(4.05%). Attack rate was higher in females 32/252 (12.7%) than in males 26/279 (9.32%), and higher 45/275(16.36%) in 3–5 years than those in 5–8 years 13/256(5.08%) children. Case fatality ratio was zero. All cases were vaccinated against measles but unvaccinated against rubella.

**Conclusions:**

Attack rate was higher in females than in males and higher in 3–5 years than 5–8 years children. We recommended establishing rubella surveillance system, conducting sero-prevalence of rubella among child bearing age females and establishing CRS surveillance among young infants to provide evidence-based information for RCV introduction. It was also recommended to develop a national rubella surveillance guideline which aid to exclude rubella cases from schools during outbreak.

**Electronic supplementary material:**

The online version of this article (10.1186/s12879-019-3873-y) contains supplementary material, which is available to authorized users.

## Background

Rubella is an acute contagious viral disease caused by a Togavirus of the genus *Rubivirus.* Rubella is a childhood disease usually having mild clinical presentations with maculopapular rash occurring in 50–80% of rubella-infected individuals. Rubella is transmitted through direct or droplet contact from nasopharyngeal secretions with an incubation period ranging from 12 to 23 days, with an average of 14 days [1, 2].

Rubella is the leading causes of birth defects worldwide [3]. Rubella infection occurring just before conception and during the first trimester of pregnancy may result in miscarriage, fetal death, premature delivery and constellation of severe birth defects called Congenital Rubella Syndrome (CRS) [1, 2, 4, 5] in up to 90% of infections [6]; though, rubella usually occurs during childhood. Worldwide, in 2010, estimates suggest that more than 100,000 babies were born with CRS [7]. Rubella is also one of the few known causes of autism [8].

Rubella-containing vaccine (RCV) prevents rubella and CRS when used in childhood immunization services and rubella-susceptible older age groups [9]. The WHO Regions of the Americas and, the European and the West pacific have set a plan to control or eliminate rubella by 2010 and 2015, respectively. However, the African region has not yet established rubella control or elimination goals [10, 11]. In Ethiopia, currently, rubella vaccine has not been included in childhood routine immunization programmes. However, in the major urban areas, some private practitioners provide RCV to children at 9 months of age or older in the form of measles-rubella vaccine; though, the coverage is unknown among the general population as the services is not monitored yet.

Surveillance is important to determine rubella incidence and mortality which are used as measures of effectiveness of rubella control and eradication strategies thereby helping to prioritize activities [12]. In Ethiopia, rubella and CRS does not have separate surveillance system, however, rubella cases are detected by measles surveillance system as suspected measles cases negative for measles IgM is tested for rubella IgM. In Ethiopia, Getahun, *et al*. [13] indicated that 18 rubella outbreaks were reported per year through measles surveillance system between 2009 and 2015 indicating that two in ten of the rubella cases were notified from Addis Ababa during the period. Yeka sub-city woreda 13 public health emergency management office notified suspected measles outbreak to Addis Ababa Health Bureau on 8 February 2018. The outbreak was later confirmed to be rubella outbreak. We investigated the outbreak to identify its etiology, describe the outbreak, and implement control measures.

## Methods

### Study area

Addis Ababa is the capital city of Ethiopia which is divided into 10 sub-cities including Yeka sub-city where the outbreak had occurred. Yeka sub-city has been further divided into 13 woredas (also called districts) and each woreda has one public health office and health center. The sub-city also has one public hospital and, four hospitals and 43 clinics run under private and non-governmental organizations. The sub-city has a total population of 433,599 and males account for 201,156(46.4%) [14].

The outbreak was occurred in a private school located in Yeka sub-city between 8 February and 20 April 2018. The school has three separate compounds within its main compound: pre-kindergarten (PKG) and kindergarten (KG) classes, 1-4 grades and 5- 10 grades. The cases were occurred only in PKG and KG classes. The school has 15 PKG and KG classes with each having an area of about 36m^2^ with an average of 35 students in each class representing nearly a student per squared kilometers (km^2^).Pre-kindergarten has seven classes (A to G). Whereas; kindergarten classes are divided into lower KG (LKG) and upper KG (UKG) classes with each LKG and UKG having four classes (A to D). The school has a total of 1431 students with PKG and KG accounting for 47.4 %( 17.26%(247) PKGs and 30.12% (284)KGs). Only 198(13.84%) of the school students use common school bus while the rest come to the school either on their feet or by their parents’ cars.

### Source population and study population

We defined the source population as students (PKG, KG, 1-4 and 5-10 students) learning in the schools of Yeka sub-city during the outbreak. We also defined the study population as students (PKG, KG, 1-4 and 5-10 students) learning in the school in which the outbreak had occurred.

### Study Design and Sampling

We described the outbreak using descriptive epidemiology. We included all suspected cases of rubella occurring during the course of the outbreak (between 8 February and 20 April, 2018) in our study.

### Operational definitions

We defined suspected cases of rubella as a student with generalized rash while a confirmed case was a suspected case tested positive for rubella IgM in the school during the outbreak. We also defined epidemiological linkage as cases occurring in the school within 30 days time frame subsequent to a laboratory confirmed case [15]. In this article, descriptive epidemiology refers to description of the outbreak in terms of person (sex, age) and time of rash onset.

### Data collection

We used structured questionnaire to collect cases information (Additional file 1). We also line listed the cases; reviewed case students’ school record and collected school information using checklists. Moreover, we reviewed the lists of the school students using the school common bus service to identify contacts in the bus. Furthermore, we interviewed parents of cases to collect information on measles vaccination and to check for any kind of vaccination the case received outside expanded program for immunization.

### Laboratory procedures

Blood samples were collected from 15 cases and transported for serological test at Ethiopia Public Health Institute Laboratory. Blood samples were tested for rubella specific IgM after they were tested negative for specific measles IgM. We used the laboratory procedures previously described by Getahun *et al*. [13] for testing the blood samples.

### Environmental investigations

We investigated the classes of the school to assess its ventilation, estimated area and number of students in each class. We also assessed risk factors that facilitate aerosol transmission among the students such as overcrowding during students’ rest or play times and using common school bus service. Moreover, we assessed health care services in the school. Furthermore, we assessed the availability of vitamin A stock and other supplies for measles/rubella case management in woreda 13 health center.

### Data analysis

We calculated age-specific and sex-specific attack rate. We also calculated percentages, means, medians, standard deviations and interquartile ranges using Epi Info™ (version 7.2.0.1).

## Results

### Descriptive epidemiology

Yeka sub-city woreda 13 public health emergency management office notified two index cases to Addis Ababa Health Bureau on 8 February, 2018. The first index case was a 4 and half year- old male student in LKG in class A. He lives in a family of 5 members in Tafo local area, Oromia. The second index case was a 5 year-old male student in UKG in class B. He lives in a family of 4 members in Abado condominium. Both index cases had no history of travel to other places, Addis Ababa 14 days before their illness onset. Families of the index cases replied that there were no an individual with rash in their vicinity 14 days before onset of illnesses of the index cases. Both of them were vaccinated for measles but they were unvaccinated against rubella. Both of them were positive for rubella IgM, however, the source of the outbreak was unknown.

**Fig. 1:**
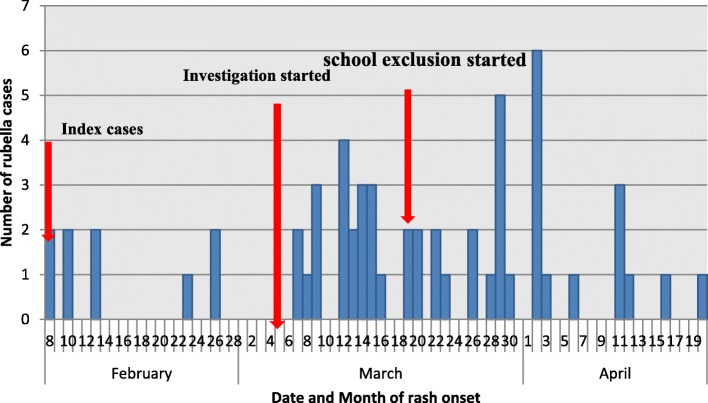
Rubella cases by date of rash onset, Yeka sub-city, Addis Ababa, Ethiopia: 8 February − 20 April, 2018

We identified 58 rubella cases during the outbreak (6 laboratory confirmed, 52 epidemiologically linked). The outbreak began on 8 February 2018 having multiple intermittent peaks during its course reaching its highest peak at 2 April, 2018 and ended on 20 April, 2018 (Fig. 1). The index cases were reported from two classes (One case from each LKG A and UKG B); however, cases were occurred in 13 (86.67%) of the 15 classes of PKGs and KGs during the course of the outbreak. Cases were not occurred in grades 1-10 classes during the outbreak. On average, nearly 2 cases were reported per day during the outbreak (mean: 2.15; 75% percentile: 3; standard deviation (SD): 1.41).

More than half 32 (55.17%) of the cases were females resulting in a female to male ratio of 1.23:1. Nearly three-quarters 45 (77.59%) of the cases were between 3 and 5 years children (median age: 4.6 years; SD: 0.87; IQR: 4-5 years). Nearly six in ten 36 (62.07%) of the cases were PKG students. (Table 1).

**Table 1:** Characteristics of rubella cases, Yeka Sub-city, Addis Ababa, Ethiopia: 8 February -20 April, 2018

Variables	Cases (*n* = 58)	
	Frequency	Percent
Sex		
Male	26	44.83%
Female	32	55.17%
Age (years)		
3–5	45	77.59%
5–8	13	22.41%
KG Level		
PKG	36	62.07%
LKG	16	27.59%
UKG	6	10.34%
Student size in the class		
25–35	22	37.93%
36–39	32	55.17%
40–42	4	6.90%
Use common school bus		
Yes	5	8.62%
No	53	91.38%

An overall attack rate was 10.92 % (58/531) in the PKG and KG students with no cases among 1-10 grades students. Attack rate was higher in females 32(12.70%) than in males (9.32%). Also, higher attack rate 45 (16.36%) was in below five years (3-5 years) children than in 5-8 years children 13 (5.08%). The highest attack rate 36 (14.57%) was among PKG students. Case fatality ratio was zero (Table 2). All cases were vaccinated against measles but not against rubella.

**Table 2:** Rubella outbreak attack rates, Yeka Sub-city, Addis Ababa, Ethiopia: 8 February − 20 April, 2018

Variable	Number of cases	population	Attack rate (%)
Age group			
3–5	45	275	16.36
5–8	13	256	5.08
Total	58	531^a^	10.92
Sex			
Male	26	279	9.32
Female	32	252	12.70
Total	58	531^a^	10.92
Grades			
PKG	36	247	14.57
LKG	16	154	10.39
UKG	6	130	4.62
Total	58	531^a^	10.92
Student size in the class			
25–35	22	221	9.95
36–39	32	268	11.94
40–42	4	42	9.52
Total	58	531^a^	10.92

^a^ refers to total students in PKG and KG excluding 1-10 grade students

### Laboratory results

Of the 15 samples tested, all were negative for measles whereas 6 cases were rubella IgM positive; 4 cases were rubella IgM negative; and 5 cases were indeterminate.

### Environmental investigation

Pre-kindergarten and kindergarten students gathered together in various occasions that could facilitate aerosol disease transmission: before class session, at their rest, at their snap time (PKG’s) and while using common bus to the school. At rest, two or more classes of the same level (example, PKG A, B, etc) could come and play together. Another occasion, for PKG students to get together is at their snap time when they sleep in mass. Lower and upper KG students take their snap at their seat in their class. However, PKG and KG students do not intermingle with grades 1 to 10 students as their compounds are separate leading to have different playing places and latrines. The entrance and exit of the school compound for grades 1 to 10 is also different from that of PKG and KG students. However, some PKG, KG and 1 to 10 grade students use school bus in common.

Moreover, we assessed the school’s health care system for the students. The school has a school nurse. The nurse has registration book to record name, age, sex, grade, class and main symptoms of sick students. Furthermore, the assessment of woreda 13 health center showed sufficient vitamin A stock availability during the outbreak.

### Public health interventions

We searched for new cases in the school on daily basis during the outbreak. Schools in the sub-city particularly those in woreda 12 and 13 were notified about the outbreak through official letter explaining their responsibility to immediately report students with signs of rash. We educated the schools teachers and other staffs on measles signs (focusing rash) so that they can identify students with rash and report to the school nurse. Students with rash were isolated and their parents were called to take them to health center for treatment and sample collection. The case students were excluded from the school for at least seven days to minimize transmission among the students. The school nurse and the local health extension workers provided education to the parents of case students to isolate the student from other individuals either within or outside the family to minimize transmission. Also, awareness creations were provided on rubella infection control and prevention specifically emphasizing its high risk among pregnant women as it causes CRS in them. So, they advised parents that family members other than pregnant women should care of the case-patients. We called the family to check the status of the case and to search for contact case in the family using their phone numbers from the school. Health extension workers were initiated to have vigilant search of new cases at their catchment areas to control the spread of the outbreak in the community.

## Discussion

We found that children between ages of 3 and 5 years were more affected than those between 5 and 8 years. The outbreak also suggested that females were more affected than males. This outbreak might be attributed to the fact that our country does not provide RCV in national immunization services making most of the children susceptible to rubella.

Our study showed that higher proportions of rubella cases were children aged between 3 and 5 years-old. This finding is similar with earlier studies in Ethiopia showing rubella outbreak occurrence in early years of age: Mitiku, *et al.* [16] indicated that 94.7% of rubella cases were <15 years-old and Getahun *et al.* [13] showed that the mean age of rubella cases were 7.3 years-old. Similar findings were also reported from a study in Kenya whereby the median age of rubella cases was 4 years-old [17] and in Nigeria whereby 58.3% of rubella cases were <5 years-old [18]. The high incidence of rubella cases in young children might be attributed to lack of acquired immunity. However, older persons could have acquired immunity due to infection at their earlier age making them resistant to rubella re-infection [19]. In our study, the median age of cases was 4 and half years. This could possibly be the factor for the occurrence of this outbreak in only PKG and KG classes but not in 1-10 grades classes.

In our investigation, we found that higher proportions of rubella cases were females. Previous studies indicated higher rubella infection in females than in males: 54% females in Ethiopia [16]; 52.2% females in Ethiopia [13]; 51% females in Benishangul-Gumuz, Ethiopia [20] and 54% in Kenya [17]. The higher attack rate in females found in our analysis also supports the fact that females are most affected by rubella than males. Investigation is needed to better understand the underlying factors for disparity of infection between sexes.

Rubella-containing vaccine (RCV) prevents rubella and CRS in children and rubella-susceptible older age groups [9] which could be presented as measles and rubella (MR); measles, mumps and rubella (MMR), or measles, mumps, rubella and varicella (MMRV) [6, 9, 21]. Integrating measles-rubella containing vaccination, and expanded program for immunization in overall health system has been recommended for measles and rubella eradication [22]. Rubella has not been given attention, though; more infants are now born with CRS each year than there are measles deaths. An increasing number of countries have introduced rubella vaccine into their schedules; nonetheless, its worldwide coverage is still less than 50% [23]. Nearly two-thirds (67.5%) of World Health Organization (WHO) Member States included RCV in their routine immunization programmes [24]*.* Among WHO African region countries, only Burkina Faso and Tanzania introduced RCV into their supplementary immunization activities [11]. However, Ethiopia has not yet introduced RCV into national routine immunization programmes which might be attributed to this outbreak as most of the children were susceptible for rubella infection. Previous studies have documented rubella outbreak in countries not introducing rubella containing vaccines in their national vaccination programs [17, 25]. This warrants the need to establish a standalone national case-based rubella surveillance system and to conduct sero-prevalence studies in women of child bearing ages to provide evidence-based information that help decide RCV introduction into national immunization programme.

Rubella virus spreads among individuals via respiratory droplets during coughing, sneezing or talking (1). Previous studies have documented outbreak of respiratory diseases such as measles and rubella in school settings [26, 27]. Outbreak response immunization is the best method for rubella outbreak control in school setting [27]; however, it was not used for the control of this outbreak as RCV has not been introduced in our national childhood immunization programme. Rubella cases were immediately excluded from the class to interrupt its transmission among the students. Nonetheless, we were unable to measure the effectiveness of this strategy as we uniformly excluded cases immediately from all classes. However, previous studies showed an 81% secondary attack rate reduction by using school exclusion control strategy [26]. We were challenged while excluding cases from the school because the country does not have school exclusion policy for cases during outbreak situation. Nonetheless, it was conducted through persuading the school on the impact of the outbreak if it would have not exercised. This necessitates the development of school exclusion policy to be used across the country during outbreaks a policy that would be used across the country during rubella outbreak in the schools to aid excluding its cases from classes upon their detection to control the spread of the outbreak.

## Conclusion

Our study showed higher proportions of rubella infection in females than in males. The study also demonstrated higher proportions of rubella cases in children aged below five years than those between 5 and 8 years old. Moreover, the study suggested the occurrence of outbreak in rubella susceptible children as they were unvaccinated against rubella. We recommended establishing rubella surveillance system, conducting cross-sectional sero-prevalence of rubella among women of child bearing age and establishing CRS surveillance among young infants to provide evidence-based information for RCV introduction and to better understand the magnitude of rubella. It was also recommended to develop a national rubella surveillance guideline which aid to exclude rubella cases from schools during outbreak immediate to their detection. Moreover, we recommended targeting below five years children and kindergartens during rubella outbreak investigation.

Our study had two main limitations. First, rubella sero-surveillance was not conducted in the school students. This might underestimate the overall attack rate. However, this might not significantly distort the finding as the investigation used a loose case definition (a student with rash). Second, we were unable to determine the risk of rubella infection in causing CRS since we had not collected data on pregnant women for logistical and financial issues. However, health extension workers were initiated to educate communities in their catchment area focusing on risk of rubella in causing CRS in pregnant women so that they can avoid contact with cases.

## Additional file

Additional file 1: Rubella_Questionnaire (DOCX 20 kb)

## Abbreviations

CRS: Congenital Rubella Syndrome
LKG: Lower Kindergarten
MMR: Measles-Mumps-Rubella
MR: Measles-rubella
PKG: Pre-kindergarten
RCV: Rubella-containing Vaccine
UKG: Upper kindergarten
