# Supplementary material for: Rubella outbreak in the school children, Addis Ababa, Ethiopia: February–April 2018
Source: BMC Infect Dis. 2019 Mar 18;19:267. doi: 10.1186/s12879-019-3873-y (PMC6423871; doi:10.1186/s12879-019-3873-y)
Supplement: Supplementary file 1 — Rubella_Questionnaire (DOCX 20 kb) [file 12879_2019_3873_MOESM1_ESM.docx]

**Questionnaires for Rubella Outbreak investigation in school children, Addis Ababa, Ethiopia: February - April, 2018**

|  | **Part 1. Interview Information** | | | | | | | | | | | | | | | | | | | | | | | | | | | | | | | | | | | | | | | | | | | | | | | | | | | | | | | | | | | | | | |
| --- | --- | --- | --- | --- | --- | --- | --- | --- | --- | --- | --- | --- | --- | --- | --- | --- | --- | --- | --- | --- | --- | --- | --- | --- | --- | --- | --- | --- | --- | --- | --- | --- | --- | --- | --- | --- | --- | --- | --- | --- | --- | --- | --- | --- | --- | --- | --- | --- | --- | --- | --- | --- | --- | --- | --- | --- | --- | --- | --- | --- | --- | --- | --- |
| 1 | Case ID | | |  | | | | | | Interview Date | | | | | | | | | | | | | | | | | | | | | | | | |  | | | | | | | | GPS coordinate | | | | | | | | | |  | | | | | | | | | | |
| 2 | Sub-city | | |  | | | | | | Woreda | | | | | | | | | | | | | |  | | | | | | | | | Kebele(ketena) | | | | | | | | | |  | | | | | | House# | | | | | | |  | | | | Phone | | |  |
| 3 | Participant type | | | | |  patient  patient family | | | | | | | | | | | | | | | | | | | | | | | | | | | | | | | | | | | | | | | | | | | | | | | | | | | | | | | | | |
|  | **Part 2. Demographic Information** | | | | | | | | | | | | | | | | | | | | | | | | | | | | | | | | | | | | | | | | | | | | | | | | | | | | | | | | | | | | | | |
|  | **Family information** | | | | | | | | | | | | | | | | | | | | | | | | | | | | | |  | | | | | | | | |  | | | | | |  | | | | | | | | |  | | | | | | | | |
| 4 | Sex | |  Male  Female | | | | | | | | | | | | | | | | | | | | | | | | | | | | Age(years) | | | | | | | | |  | | | | | |  | | | | | | | | | | | | | | | | | |
| 5 | Marital status | | | | | |  single  married  widowed  Divorced  separated  Others: | | | | | | | | | | | | | | | | | | | | | | | | | | | | | | | | | | | | | | | | | | | | | | | | | | | | | | | | |
| 6 | Education | | | | | | | |  | | | | | | | | | | | | | | | | | | | | | | | | | | | | | | | | | | | | | | | | | | | | | | | | | | | | | | |
| 7 | Occupation | | | | | |  | | | | | | | | | | | | | | | | | | | | | | | | | | | | | | | | | | | | | | | | | | | | | | | | | | | | | | | | |
| 8 | Number of persons in house | | | | | | | | | | | | | | | | |  | | | | | | | | | | | | | | | | | | | How many below 5 years children are there? | | | | | | | | | | | | | | | | | | | | |  | | | | | |
| 9 | Religion | | | |  | | | | | | | | | | | | | | | | | | | | | | | | | | | | | | | | | | | | | | | | | | | | | | | | | | | | | | | | | | |
| 10 | Is there any sick person with rash in the family before your illness? | | | | | | | | | | | | | | | | | | | | | | | | | | | | | | | | | | | | | | |  Yes  No | | | | | | | | | | | | | | If yes, how many? | | | | | | | |  | |
| 11 | Is there any sick person with rash in the family after your illness? | | | | | | | | | | | | | | | | | | | | | | | | | | | | | | | | | | | | | | |  Yes  No | | | | | | | | | | | | | | If yes, how many? | | | | | | | |  | |
|  | **Patient information** | | | | | | | | | | | | | | | | | | | | |  | | | | | | | | | | | | | | | | | | | | | | | | | | | | | | | | | | | | | | | | | |
| 12 | Name | | | | | | |  | | | | | | | | | | | | | | | | | | | | | | | | | | | | | | | | | | | | | | | | | | | | | | | | | | | | | | | |
| 13 | Sex |  Male  Female | | | | | | | | | | | | | | | | | | | | | | | | | | | | | | | | Age(years) | | | | | | |  | | | | | | | | | | Age(Months) | | | | | | | |  | | | | |
| 14 | Grade | | | | | | | | | | | | | |  pre-kindergarten  kindergarten  1-4 grades  5-10 grades | | | | | | | | | | | | | | | | | | | | | | | | | | | | | | | | | | | | | | | | | | | | | | | | |
| 15 | Student size in class | | | | | | | | | | | | | |  | | | | | | | | | |  | | | | | | | | | | | | | | | | | | | | | | | | | | | | | | | | | | | | | | |
|  | **Part 3. Details about illness** | | | | | | | | | | | | | | | | | | | | | | | | | | | | | | | | | | | | | | | | | | | | | | | | | | | | | | | | | | | | | | |
| 16 | Date of illness onset | | | | | | |  | | | | | | | | | | | | | | | Date of rash onset | | | | | | | | | | | | | | | | | | |  | | | | | | | | Duration of rash | | | | | | | | | |  | | | |
| 17 | Date seen at health facility | | | | | | | | | | | | | | |  | | | | | | | | | | Duration of illness before visiting health facility(in days or hours) | | | | | | | | | | | | | | | | | | | | | | | | | | | | | | | | | | |  | | |
| 18 | Place where rash started? | | | | | | | | | | | | | woreda | | | | | | | | | | | | | | | | |  | | | | | | | Ketena/kebele | | | | | | | | | |  | | | | | | | | |  Same as permanent address | | | | | | |
| 19 | Admitted? | | | | | | | | | |  Yes  No | | | | | | | | | | | | | | | | | | | | | | | | | status | | | | | | | |  Alive  Died | | | | | | | | | | | | | | | | | | | |
| 20 | Were you treated | | | | | | | | | | | |  Yes  No | | | | | | | | | | | | | | | | | | | | | | | | | | | | | | | | | | | | | | | | | | | | | | | | | | |
| 21 | If yes, which of these (tick all that applies)? | | | | | | | | | | | | | | | | | | | | | | | | | | |  ORS  Antibiotics  Vitamin A  Supplementary food  TTC ointment  Anti Pyretic  Others: | | | | | | | | | | | | | | | | | | | | | | | | | | | | | | | | | | | |
| 22 | Response to treatment? | | | | | | | | | | | | | | | | | | |  cured  partial  deteriorated/disabled  death | | | | | | | | | | | | | | | | | | | | | | | | | | | | | | | | | | | | | | | | | | | |
| 23 | Which symptoms/signs (tick all that applies)?? | | | | | | | | | | | | | | | | | | | | | | | | | | | |  Fever  Rash  Cough  Coryza (runny nose)  Conjunctivitis (red eyes)  Ear discharge   Pneumonia  Vomiting  Others: | | | | | | | | | | | | | | | | | | | | | | | | | | | | | | | | | | |
| 24 | Which complications? | | | | | | | | | | |  Pneumonia  Diarrhea  Otitis media (ear discharge)  Convolution  Corneal drying  Blindness  Feeding problem  Others: | | | | | | | | | | | | | | | | | | | | | | | | | | | | | | | | | | | | | | | | | | | | | | | | | | | |
|  | **Part 4: Vaccination** | | | | | | | | | | | | | | | | | | | | | | | | | | | | | | | | | | | | | | | | | | | | | | | | | | | | | | | | | | | | | | |
| 25 | Were you vaccinated for measles? | | | | | | | | | | | | | | | | | |  Yes  No  Unknown  Not applicable | | | | | | | | | | | | | | | | | | | | | | | | | | | | | | | | | | | | | | | | | | | | |
| 26 | If yes, last vaccination date(day-month-year) | | | | | | | | | | | | | | | | | | | | | | | | | | | | |  Patient recall_________________  Vaccination card____________  Don’t remember | | | | | | | | | | | | | | | | | | | | | | | | | | | | | | | | | |
| 27 | Number of vaccine doses received | | | | | | | | | | | | | | | | | | | | | | | | | |  One dose  Two dose  Three and above  Don’t remember | | | | | | | | | | | | | | | | | | | | | | | | | | | | | | | | | | | | |
| 28 | Age at first vaccination? | | | | | | | | | | | | | | | | Age(years) | | | | | | | | | | | | | | |  | | | | | | | Age(Months) | | | | | | | | | | | | |  | | | | | | | | | | | |
| 29 | If not vaccinated why? | | | | | | | | | | | | | | | | | |  lack of knowledge about vaccination  absence during vaccination campaign  other(specify): | | | | | | | | | | | | | | | | | | | | | | | | | | | | | | | | | | | | | | | | | | | | |
| 30 | Were you vaccinated for rubella? | | | | | | | | | | | | | | | | | |  Yes  No  Unknown  Not applicable | | | | | | | | | | | | | | | | | | | | | | | | | | | | | | | | | | | | | | | | | | | | |
|  | **Part 5: Travel and contact History** | | | | | | | | | | | | | | | | | | | | | | | | | | | | | | | | | | | | | | | | | | | | | | | | | | | | | | | | | | | | | | |
| 31 | Did you travel to areas with active rash diseases 14 days before onset of your illness? | | | | | | | | | | | | | | | | | | | | | | | | | | | | | | | | | | | | | | | | | | | |  Yes  No  Don’t remember | | | | | | | | | | | | | | | | | | |
| 32 | If yes, where? | | | | | | | | | | | | | | | | | | | |  | | | | | | | | | | | | | | | | | | | | | | | | | | | | | | | | | | | | | | | | | | |
| 33 | Did you contact with a person having rash symptoms 14 days before onset of your illness? | | | | | | | | | | | | | | | | | | | | | | | | | | | | | | | | | | | | | | | | | | | | | |  Yes  No  Don’t remember | | | | | | | | | | | | | | | | |
